# Rapid detection of multidrug resistance in tuberculosis using nanopore-based targeted next-generation sequencing: a multicenter, double-blind study

**DOI:** 10.3389/fmicb.2024.1349715

**Published:** 2024-03-01

**Authors:** Aimei Liu, Sang Liu, Kangyan Lv, Qingdong Zhu, Jun Wen, Jianpeng Li, Chengyuan Liang, Xuegang Huang, Chunming Gong, Qingfeng Sun, Hongcang Gu

**Affiliations:** ^1^Department of Tuberculosis, Guangxi Zhuang Autonomous Region Chest Hospital, Liuzhou, Guangxi, China; ^2^Department of Tuberculosis, The Fourth People's Hospital of Nanning, Nanning, Guangxi, China; ^3^Department of Pulmonary Medicine, The Third People's Hospital of Guilin, Guilin, Guangxi, China; ^4^Department of Pulmonary Medicine, The Third People's Hospital of Wuzhou, Wuzhou, Guangxi, China; ^5^Department of Infectious Diseases, The People's Hospital of Baise, Baise, Guangxi, China; ^6^Department of Infectious Diseases, The First People's Hospital of Fangchenggang, Fangchenggang, Guangxi, China; ^7^Institute of Health and Medical Technology, Hefei Institutes of Physical Science, Chinese Academy of Sciences, Hefei, Anhui, China

**Keywords:** *Mycobacterium tuberculosis*, drug-resistant tuberculosis, nanopore sequencing, mutation, molecular diagnosis

## Abstract

**Background:**

Resistance to anti-tuberculous drugs is a major challenge in the treatment of tuberculosis (TB). We aimed to evaluate the clinical availability of nanopore-based targeted next-generation sequencing (NanoTNGS) for the diagnosis of drug-resistant tuberculosis (DR-TB).

**Methods:**

This study enrolled 253 patients with suspected DR-TB from six hospitals. The diagnostic efficacy of NanoTNGS for detecting *Mycobacterium tuberculosis* and its susceptibility or resistance to first- and second-line anti-tuberculosis drugs was assessed by comparing conventional phenotypic drug susceptibility testing (pDST) and Xpert MTB/RIF assays. NanoTNGS can be performed within 12 hours from DNA extraction to the result delivery.

**Results:**

NanoTNGS showed a remarkable concordance rate of 99.44% (179/180) with the culture assay for identifying the *Mycobacterium tuberculosis* complex. The sensitivity of NanoTNGS for detecting drug resistance was 93.53% for rifampicin, 89.72% for isoniazid, 85.45% for ethambutol, 74.00% for streptomycin, and 88.89% for fluoroquinolones. Specificities ranged from 83.33% to 100% for all drugs tested. Sensitivity for rifampicin-resistant tuberculosis using NanoTNGS increased by 9.73% compared to Xpert MTB/RIF. The most common mutations were S531L (codon in E. coli) in the *rpoB* gene, S315T in the *katG* gene, and M306V in the *embB* gene, conferring resistance to rifampicin, isoniazid, and ethambutol, respectively. In addition, mutations in the pncA gene, potentially contributing to pyrazinamide resistance, were detected in 32 patients. Other prevalent variants, including D94G in the *gyrA* gene and K43R in the *rpsL* gene, conferred resistance to fluoroquinolones and streptomycin, respectively. Furthermore, the *rv0678* R94Q mutation was detected in one sample, indicating potential resistance to bedaquiline.

**Conclusion:**

NanoTNGS rapidly and accurately identifies resistance or susceptibility to anti-TB drugs, outperforming traditional methods. Clinical implementation of the technique can recognize DR-TB in time and provide guidance for choosing appropriate antituberculosis agents.

## 1 Introduction

Recent years, the COVID-19 pandemic has posed a major concern for global public health, including the rising prevalence and delayed diagnosis in tuberculosis (TB; Pai et al., 2022). The World Health Organization (WHO) Global Tuberculosis Report 2022 revealed increasing TB cases, with more than 10 million individuals affected. In 2021 alone, approximately half a million new cases with rifampicin-resistant tuberculosis (RR-TB) were reported, and 78% of them were multidrug-resistant tuberculosis (MDR-TB; Finci et al., 2022). However, only one-third of patients diagnosed with multidrug-resistant or rifampicin-resistant TB can get effective treatment (Zürcher et al., 2021; World Health Organization, 2022). Meanwhile, the transmission of drug-resistant *Mycobacterium tuberculosis* (*M.tb*) between people has complicated the treatment and prevention of TB. Rapid prediction of drug resistance to *Mycobacterium tuberculosis* and administering appropriate antibiotics to patients remain high priorities for effective control of drug-resistant tuberculosis (DR-TB; Wells et al., 2013).

Phenotypic drug susceptibility testing (pDST) is currently the “gold standard” for determining antibiotic resistance (Votintseva et al., 2017). This method depends on the bacterial culture and usually requires a couple of weeks to get final results. Moreover, technical requirements also pose some difficulties during the laboratory procedures and give patients inadequate access to pDST-based drug-resistance detection. With the great development of high-throughput sequencing technologies such as next-generation sequencing (NGS) and Oxford nanopore sequencing, rapid molecular or genotypic drug susceptibility testing was more widely used for selecting appropriate antibiotic treatments and became an important complement to pDST (Gliddon et al., 2021; Zhao et al., 2022; Hall et al., 2023; Jouet et al., 2023). Xpert MTB/RIF is one of the WHO-endorsed rapid nucleic acid amplification tests for the detection of *Mycobacterium tuberculosis* and rifampicin (RIF) resistance in 2 h (Yu et al., 2022). However, Xpert MTB/RIF is limited to the detection of high-prevalence mutations in the 81-bp rifampin resistance determining region (RRDR) of the *rpoB* gene (Votintseva et al., 2017; Tafess et al., 2020). A diagnostic test covering a broader range of resistance to all currently used anti-TB medications was needed in the clinical setting to rapidly detect the multidrug-resistant pattern.

Previous studies showed that targeted NGS was widely used in clinical practices to identify drug resistance in isolates (Tafess et al., 2020; Walker et al., 2022). Nanopore-based targeted next-generation sequencing (NanoTNGS) is developed on the Oxford nanopore sequencing platform specifically for the detection of mycobacterial species and the prediction of drug resistance in *Mycobacterium tuberculosis* with a longer read length. NanoTNGS can simultaneously detect resistances to both first- and second-line drugs, which were conferred by known and uncharacterized mutations. The amount of DNA required and the interference from host DNA sequences are reduced by amplifying targeted gene regions prior to sequencing (Jouet et al., 2021). However, its ability to identify the resistance or susceptibility of *Mycobacterium tuberculosis* directly from clinical specimens remains to be elucidated.

In this study, we systematically evaluated the diagnostic performance of genotypic drug susceptibility testing using NanoTNGS compared with pDST and Xpert MTB/RIF for identifying resistance to anti-TB drugs.

## 2 Materials and methods

### 2.1 Study design and population

This prospective multicenter, cross-sectional study enrolled six hospitals located in the Guangxi Zhuang Autonomous Region, demonstrating a high TB incidence in China (Yang et al., 2022). The six enrolled hospitals were as follows: the Guangxi Chest Hospital, the Fourth People’s Hospital of Nanning, the Third People’s Hospital of Guilin, the First People’s Hospital of Fangchenggang, the Third People’s Hospital of Wuzhou, and Baise People’s Hospital.

In total, 253 patients were screened in this study from those hospitals between January 2022 and July 2023. NanoTNGS assays were performed directly from the clinical specimens among all the participants. Other eligibility criteria include a positive culture result or a positive result from Xpert MTB/RIF.

### 2.2 Sample collection and DNA extraction

We collected 253 specimens in sterile specimen collection tubes from patients with suspected DR-TB for laboratory testing at the participating hospitals. Several laboratory methods were employed, including the acid-fast bacillus (AFB) smear, Xpert MTB/RIF, NanoTNGS, culture-based *Mycobacterium* identification, and pDST, depending on specific laboratory tests used in different hospitals. A total of 184 samples from the screened population met the eligibility criteria. Patient information, including gender, age, body mass index (BMI), nationality, previous therapeutic regimen of anti-TB drugs, smoking history, and symptoms, were also collected. The results of NanoTNGS were analyzed by individuals who were blind to the pDST and Xpert MTB/RIF results. The laboratory test results were confirmed with the electronic medical record in each hospital at the end of the study.

Sputum samples were decontaminated, liquefied, and lysed using N-acetyl-L-cysteine (NALC)-NaOH at a final concentration of 2% (Leung et al., 2022). The bronchoalveolar lavage fluid (BALF) samples were then centrifuged at low speed (3,000 rpm for 10 min) to enrich the cells. The enriched cells were then adequately ground and digested with lysozyme according to standard laboratory procedures. After sample pre-treatment, DNA extraction was performed using the QIAamp DNA Microbiome Kit (Cat. No. 51707, Qiagen, Hilden, Germany) according to the manufacturer’s protocol. Blank EB buffer was used as a negative control for nucleic acid extraction. The concentration of extracted DNA was measured using the Invitrogen Qubit 4 Fluorometer and then stored at −20°C for subsequent library preparation.

### 2.3 Multiplex PCR, library preparation, and sequencing

The library preparation for the NanoTNGS assay involved a two-step polymerase chain reaction (PCR) process. A total volume of 30 μL of PCR mixture included 50 ng DNA extract, 3 μL of primers with a concentration of 0.2 pmol/μL, and 15 μL of Multiplex PCR Master Mix (Cat. No. BR0200801, Biotechrabbit GmbH, Berlin, Germany) during the first PCR step. The primers were designed to target *16S rDNA* and *hsp65* for the identification of *Mycobacterial* species and *rpoB*, *katG*, *thyA*, *ahpC*, *alr*, *eis*, *embA*, *embB*, *ethA*, *folC*, *gibB*, *gyrA*, *gyrB*, *inhA*, *pncA*, *rplC*, *rpsL*, *rrs*, *rv0678*, *atpE*, and *tlyA* for the prediction of resistance genes. The types of anti-TB drugs that correspond to the resistant gene regions are shown in Supplementary Table 1. The amplifying reaction was carried out as follows: pre-denaturation at 95°C for 3 min, followed by 35 cycles of denaturation at 95°C for 15 s, annealing at 60°C for 45 s, extension at 72°C for 15 s, and a final extension at 72°C for 3 min.

The products obtained from the first amplification step were then subjected to Nanopore PCR Barcode Expansion using the same Multiplex PCR Master Mix. The reaction conditions for this step were pre-denaturation at 95°C for 3 min, followed by 35 cycles of denaturation at 95°C for 30 s, annealing at 64°C for 30 s, extension at 72°C for 1 min, and a final extension at 72°C for another 3 min.

To assess the concentration of the purified library after this process, we used the Equalbit 1 × dsDNA HS Assay Kit (Cat. No. EQ121-01, Nanjing Vazyme Biotech, Nanjing, CN). An end-tail repair was performed using the End Prep Mix 4 from the VAHTS Universal End Preparation Module for Illumina V2 (Cat. No. N203, Nanjing Vazyme Biotech, Nanjing, CN). Adapter ligation was carried out using the Rapid DNA Ligase and Rapid Ligation Buffer from the VAHTS Universal DNA Library Prep Kit for Illumina V3 (Cat. No. ND607, Nanjing Vazyme Biotech, Nanjing, CN). Ligation Sequencing Kit V14 (SQK-LSK114, Oxford Nanopore Technologies, Oxford, United Kingdom) was employed to construct the pooled libraries. Following each amplification step, the VAHTS DNA Clean Beads (Cat. No. N411-01, Nanjing Vazyme Biotech, Nanjing, CN) were utilized for purification.

Finally, 12 μL of the pooled library was loaded into a nanopore flow cell (R9.4.1) for sequencing on the GridION sequencing platform. Base calling data were generated using MinKNOW version 2.0, and reads of small size and/or low quality were removed using NanoFilt. *Mycobacterium tuberculosis* was considered positive when at least one read was mapped to either the species or genus level (Miao et al., 2018). Antibiotic-resistance genes detected by targeted nanopore sequencing were predicted using the Comprehensive Antibiotic Resistance Database (CARD), the ResFinder, or the TBProfiler database with default alignment settings (≥80% identity over ≥60% of the length of the target gene; Yonkus et al., 2022). Thresholds for variant calling were set at allele frequency greater than 10%, depth greater than 10, and allele depth greater than 5 (CRyPTIC Consortium and the 100,000 Genomes Project et al., 2018).

### 2.4 Culture, acid-fast bacillus microscopy, and phenotypic drug susceptibility testing

The sputum and BALF were pre-treated with NALC-NaOH and subjected to vortex oscillation. The suspension was adjusted to a final concentration of 2%. A 100 μL of suspension was then incubated on the upper and middle parts of the Löwenstein–Jensen agar slant for up to 8 weeks. Slides prepared for direct microscopy were stained with the Ziehl–Neelsen method after heat fixation and were evaluated semi-quantitatively as negative, +, ++, and +++ (Pfyffer, 2015). Heparin was added to the hydrothorax and cerebrospinal fluid for anticoagulation, but no other pre-treatment was performed prior to DNA extraction, culture, and microscopy.

Suspensions of isolates from positive samples were incubated on 7H10 agar using an agar proportion assay, following the methods of the Clinical and Laboratory Standards Institute (CLSI). The pDST was carried out on drugs including RIF, isoniazid, ethambutol (EMB), streptomycin, kanamycin, amikacin, capreomycin, moxifloxacin, or levofloxacin. In cases where the laboratory tests yielded negative cultures, a recent, preceding DST was used for comparison with the NanoTNGS results.

### 2.5 Statistical analyses

The sensitivity of each drug was determined by computing the ratio of samples identified as resistant by the pDST, and the samples were also identified as resistant by the Xpert MTB/XDR or NanoTNGS assay (Penn-Nicholson et al., 2022). The specificity was computed by the ratio of samples identified as susceptible according to both the pDST and Xpert MTB/XDR results. Continuous data without normal distribution were presented using medians and interquartile ranges (IQRs), while categorical variables were described using proportions. All analyses were conducted using IBM SPSS Statistics 24.

## 3 Results

### 3.1 Patients characteristics

**Figure 1:**
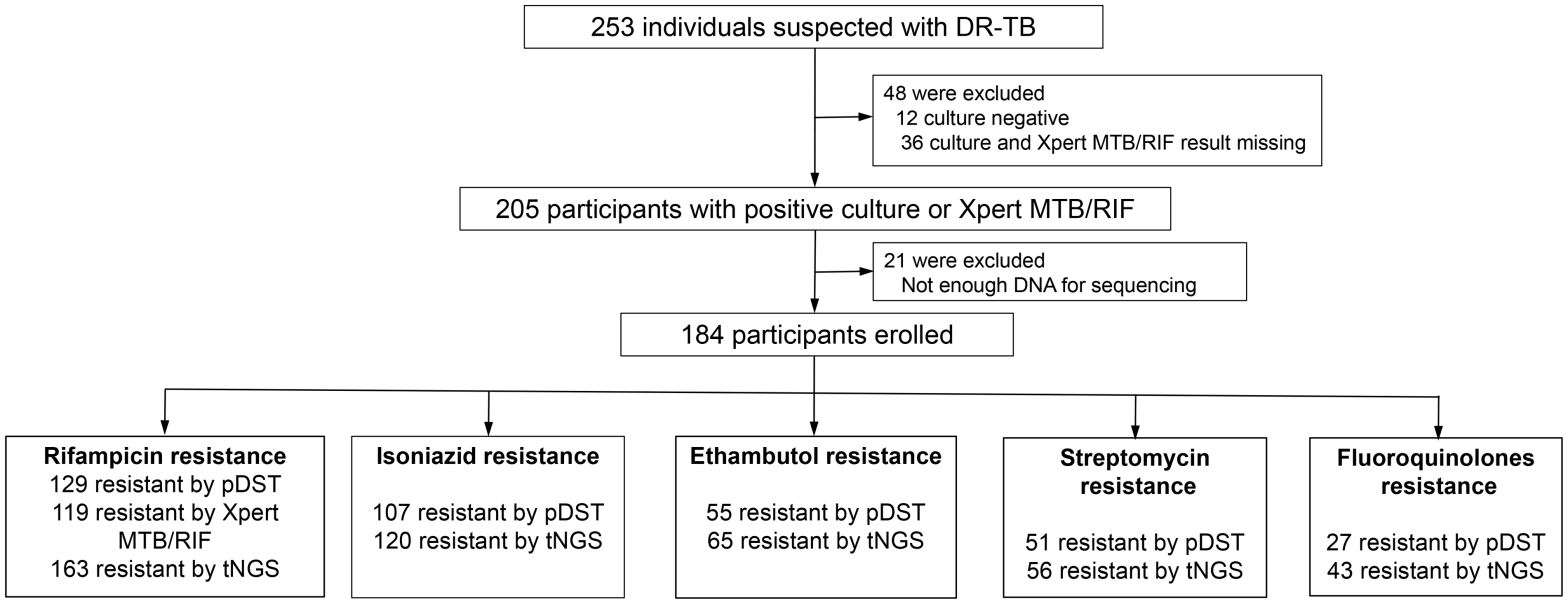
A flowchart of study and diagnostic classifications of drug-resistant patients. DR-TB, drug-resistant tuberculosis. pDST, phenotypic drug susceptibility testing. AFB, acid-fast bacilli smear. NanoTNGS, nanopore-based targeted next-generation sequencing.

During the period between January 2022 and July 2023, a total of 253 suspected DR-TB patients were screened for eligibility. A total of 69 participants were excluded from the downstream analysis mainly due to negative culture tests, missing culture and Xpert MTB/RIF results or low quality of genomic DNA extraction (Figure 1). Only samples with positive culture results will be subjected to subsequent phenotypic drug susceptibility tests to compare with the prediction of resistance by NanoTNGS. Therefore, we excluded 48 cases of culture-negative or result-missing results. Finally, 184 patients were enrolled in the study. The samples included 90 (48.91%) BALF, 89 (48.37%) sputum, 2 mixed BLAF and sputum (0.59%), and 3 (1.63%) other sample types (hydrothorax, ascites, and pus).

The median age of the patients was 48 years, with 77.13% being male individuals and 15.21% being current smokers. The Han population accounted for the majority at 65.76%, followed by Zhuang people at 29.35%, and other ethnic minorities, including Miao, Dong, Yao, Maonan, and Tibetan, at a combined percentage of 4.89%. Among the enrolled participants, 46.20% (85 out of 184) were underweight with a BMI below <18.5, indicating increased nutrient consumption needs. The underweight population, as defined by BMI, was associated with a higher risk of TB incidence (Cho et al., 2022). These participants exhibited typical symptoms associated with TB, such as cough (79.35%), expectoration (58.15%), fever (9.24%), and hemoptysis (7.61%). All the patients presented imaging evidence of TB infection: cavity findings in 52.17% cases, calcification in 15.76%, pleural thickening and adhesion in 27.17%, fibrosis in 26.63%, and patchy, strip-like shadows in 2.17%. The results of the erythrocyte sedimentation rate and C-reactive protein demonstrated a possible inflammation reaction (Table 1).

**Table 1:** Characteristics of patients with drug-resistant tuberculosis enrolled in this study.

Characteristics	Number
Age (Median, IRQ)	48 (37, 57)
**Gender**	
Male	142 (77.13%)
Female	42 (22.87%)
BMI (Median, IRQ)	19.03 (17.26, 21.09)
BMI < 18.5	85 (46.20%)
**Symptoms**	
Cough	146 (79.35%)
Expectoration	107 (58.15%)
Fever	17 (9.24%)
Hemoptysis	14 (7.61%)
Previous treatment of tuberculosis infection	98 (53.26%)
Erythrocyte Sedimentation Rate (mm/h)	50.00 (28.00, 85.90)
C-reactive protein (mg/L)	31.41 (10.90, 75.50)
**Image features**	
Cavity	96 (52.17%)
Calcification	29 (15.76%)
Pleural thickening adhesion	50 (27.17%)
Fibrosis	49 (26.63%)
Patchy or strip-like shadows	4 (2.17%)

Values are no. (%) except as indicated.

### 3.2 Detection for MTBC and drug resistance

**Figure 2:**
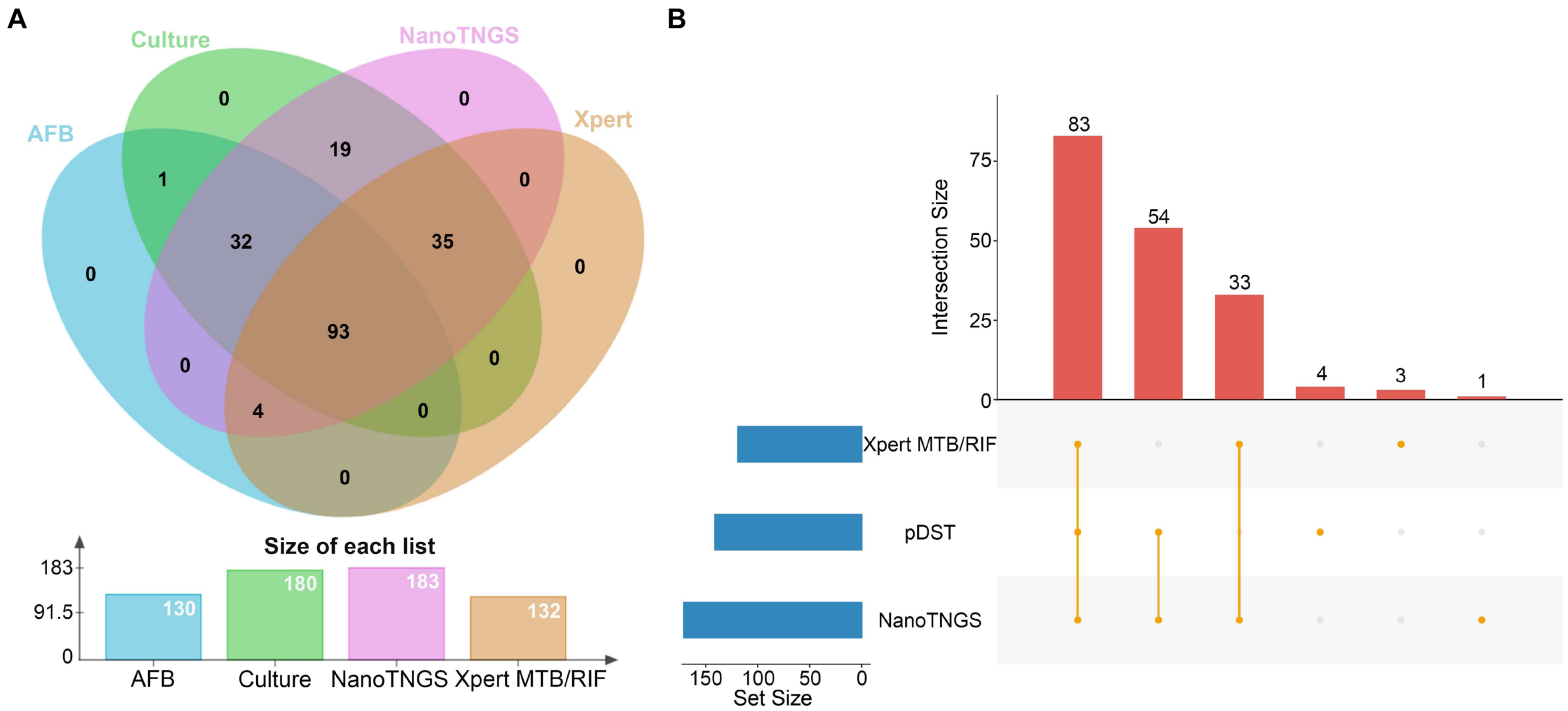
Positive case by different tests. **(A)** *Mycobacterium tuberculosis* was tested as positive. The x-axis represents the detection method, and the y-axis represents the number of positive results. **(B)** The UpSet plot showing the shared drug-resistance specimens among NanoTNGS, pDST, and Xpert MTB/RIF.

Of these enrolled participants, 130 (86.67%) out of 150 were smear positive for TB, and 180 (97.83%) out of 184 were Mycobacterium tuberculosis complex (MTBC) culture positive (Figure 2A). There were four samples that did not grow in the culture medium but were detected as positives via AFB, NanoTNGS, and Xpert MTB/RIF. Two of four patients have a history of anti-TB treatment, which may lead to false-negative culture results. Other factors contributing to *Mycobacterium tuberculosis* culture-negative results include low bacterial load, delays in specimen processing, and decontamination with NaOH (Traore et al., 2006; Madico et al., 2019). NanoTNGS identified 183 (99.46%) MTBC-positive cases from 184 samples. The positive number detected by Xpert MTB/RIF was 132 (88.00%, 132/150). For the one NanoTNGS missed sample, MTBC cases were identified via culture and AFB smear microscopy. Overall, NanoTNGS assays detected 179 MTBC positive cases among 180 samples (99.44%) confirmed by the MTBC culture, demonstrating a high level of concordance between the two methods.

There were 171, 141, and 119 drug-resistant samples detected by NanoTNGS, pDST, and Xpert MTB/RIF, respectively (Figure 2B). In four cases, drug resistance was confirmed only through pDST, while NanoTNGS did not detect drug-resistance mutations in these cases. This aspect might be because of the low level of drug-resistant gene variation. The consumable cost of NanoTNGS was approximately RMB¥ 800–900 per sample. The turnaround time for NanoTNGS was 12 h from DNA extraction to test result delivery, at least 6 weeks earlier than pDST. The workflow of NanoTNGS is shown in Supplementary Figure 1.

### 3.3 Resistant mutations for genotypic DST

The drug-related resistant gene mutations were detected by NanoTNGS directly from clinical specimens. Resistance genes were found in 171 patients for the following drugs: RIF (163 cases), isoniazid (120 cases), EMB (65 cases), streptomycin (56 cases), fluoroquinolones (43 cases), pyrazinamide (32 cases), aminoglycosides (kanamycin, amikacin, capreomycin, 5 cases), and bedaquiline (1 case). The average coverage depth of resistance genes ranged from 295× to 1,777× across 10 common gene regions detected by NanoTNGS (Supplementary Figure 2). The coverage depth of each sample is shown in Supplementary Table 2.

**Figure 3:**
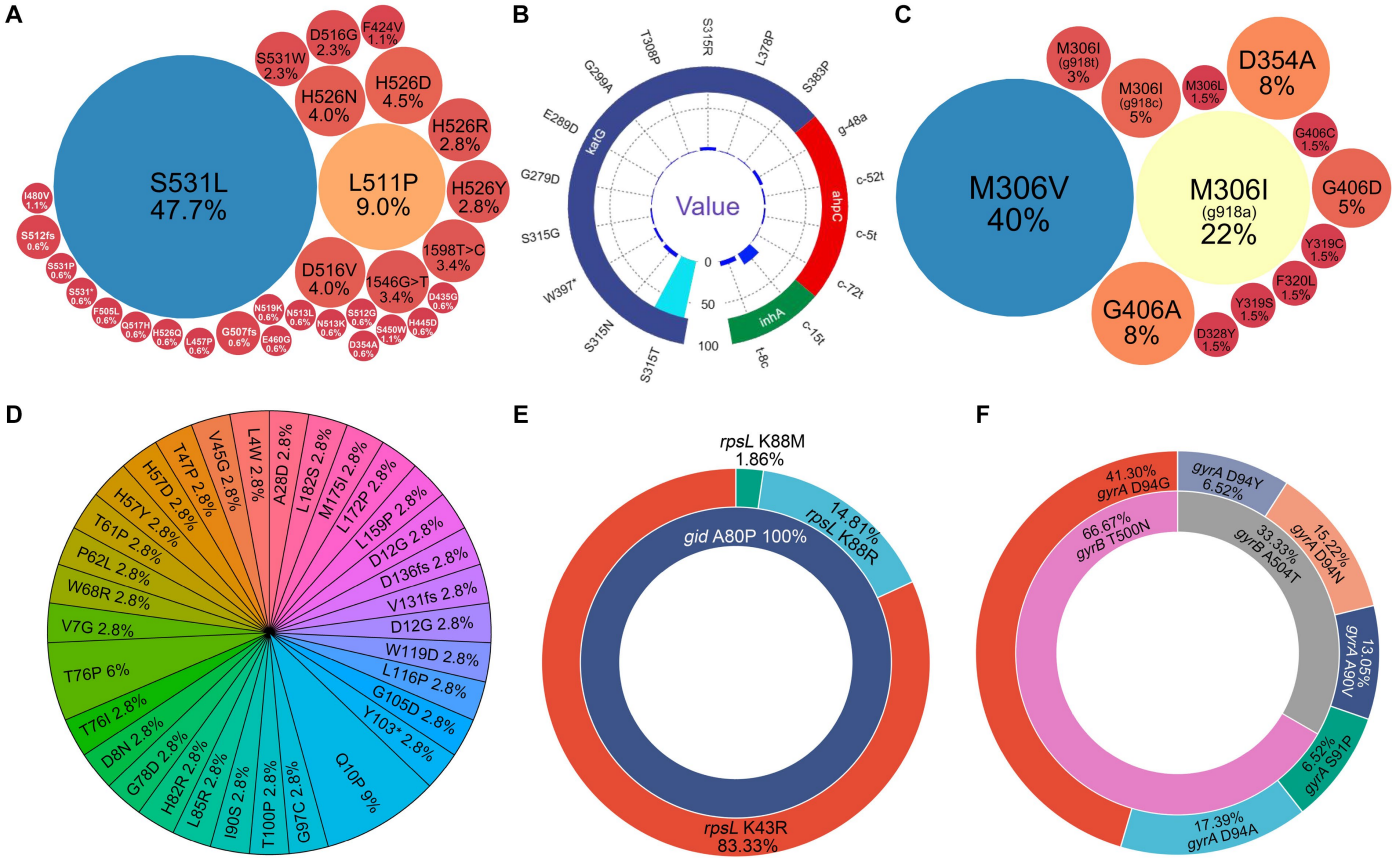
Diversity of drug-resistance gene variants associated with anti-tubercular agents predicted by NanoTNGS. **(A)** *rpoB* mutations associated with rifampicin resistance. **(B)** *katG*, *ahpC*, and *inhA* mutations associated with isoniazid resistance. **(C)** *embB* mutations associated with ethambutol resistance. **(D)** *pncA* mutations associated with pyrazinamide resistance. **(E)** *rpsL*, *rrs* mutations associated with streptomycin resistance. **(F)** *gyrA*, *gyrB* mutations associated with fluoroquinolone resistance.

In total, 176 *rpoB* gene mutations associated with potential RIF resistance were identified from the 163 samples by NanoTNGS. Among those mutations, 18 samples had multiple *rpoB* gene mutations, suggesting that different drug-resistance mechanisms may exist. The most frequent mutation was *rpoB* S531L, which accounted for 47.7% (84/176) gene mutations (Figure 3A). Other frequently observed mutations included L511P (9%, 16/176), D516V (4%, 7/176), H526N (4%, 7/176), and H526D (4.5%, 8/176).

The previous study showed that the *katG* gene at position p.S315T was associated with high-level isoniazid resistance (Finci et al., 2022). In the present study, we identified 134 gene mutations related to isoniazid resistance from 120 samples. The most common mutation was *katG* S315T, which was observed in 84 of 134 samples (Figure 3B). Mutations located upstream of the *inhA* and *ahpC* promoters were also detected. The most common upstream mutations (c-15 t and t-8c) in the *inhA* promoter region were observed in 21 of the 120 resistant isoniazid samples.

Relatively fewer resistance mutations were detected for EMB (*n* = 65), fluoroquinolones (*n* = 49), and streptomycin (*n* = 56). The most common mutations in the EMB-resistance gene were *embB* M306V (26/65) and M306I (19/65; Figure 3C). Meanwhile, 32 patients carried 35 pyrazinamide resistance-conferring *pncA* gene mutations. The mutations were dispersed in the *pncA* gene regions. The detailed gene mutation locus is shown in Figure 3D. The most common resistance gene mutation related to streptomycin was *rpsL* K43R (Figure 3E). For fluoroquinolones, the most frequent mutations were *gyrA* D94G (19/46) and D94A (8/46; Figure 3F). There were two *gyrB* T500N mutations and one *gyrB* A504T mutation potentially informing fluoroquinolone resistance. The depth, allele depth, and allele frequency of the top three variants in resistance genes detected via NanoTNGS are shown in Supplementary Table 3. The minimum average allele depth was 75.5×, which is greater than the 5× cutoff for variant call (Supplementary Figure 3). In addition, there were five *rrs* gene mutations that were associated with amikacin, kanamycin, and capreomycin resistance in five samples. One *Rv0678* D94Q mutation was detected by the NanoTNGS assay, indicating potential resistance to bedaquiline (Supplementary Table 4). Identification of resistance gene mutations by NanoTNGS allows comprehensive profiling of the resistance mechanism and interpretation of genotypic DST results.

### 3.4 Diagnostic performance of NanoTNGS for drug-resistance detection

RIF-resistance prediction achieved a sensitivity of 93.53% compared to the composite reference standard (CRS), while Xpert MTB/RIF-resistance prediction had a lower sensitivity of 83.80%. The sensitivity of NanoTNGS for predicting resistance was 89.72% for isoniazid, 85.45% for EMB, 75.22% for streptomycin, and 88.89% for fluoroquinolones, respectively, compared to pDST (Table 2).

**Table 2:** Performance of NanoTNGS in the diagnosis of drug-resistant tuberculosis compared with phenotypic drug susceptibility testing.

Drugs	TP	FP	FN	TN	Sensitivity	Specificity	PPV	NPV	TCR
RIF-NanoTNGS^#^	159	0	11	8	93.53%	100%	100%	42.11%	95.51%
RIF-Xpert^#^	119	0	23	8	83.80%	100%	100%	25.80%	84.67%
Isoniazide	96	9	11	45	89.72%	83.33%	91.43%	80.36%	87.58%
Ethambutol	47	11	8	93	85.45%	89.42%	81.03%	92.08%	88.05%
Streptomycin	37	8	13	102	74.00%	92.73%	82.22%	88.70%	86.88%
Fluoroquinolones	24	12	3	121	88.89%	90.98%	66.67%	97.58%	90.63%

^#^Diagnostic performance of the NanoTNGS or Xpert MTB/RIF assays for the detection of rifampicin resistance compared to a composite reference standard. The results of pDST and Xpert MTB/RIF were treated as a composite reference standard in evaluating the diagnostic performance. RIF, rifampicin. TP, true positive; FP, false positive; FN, false negative; TN, true negative. PPV, positive predictive value; NPV, negative predictive value; TCR, total coincidence rate.

The specificity for NanoTNGS-resistance detection was high for streptomycin (92.59%), fluoroquinolones (91.67%), EMB (89.42%), and for isoniazid (84.91%). For RIF, NanoTNGS achieved a specificity of 100% compared with CRS. Overall, the NanoTNGS achieved high specificity when detecting resistance genes.

## 4 Discussion

The traditional methods used to test the susceptibility of *Mycobacterium tuberculosis* to drugs included sample collection, laboratory procedures, and the final report of pDST, which usually took several weeks. This lengthy turnaround time limits the availability of treatment guidance and hinders the management of DR-TB in clinical practice. There is an urgent need for rapid and accurate diagnostic tools that can identify *Mycobacterium tuberculosis* and detect common mutations associated with drug resistance against anti-tubercular agents. The application of NGS for detecting drug resistance in *Mycobacterium tuberculosis* has been proven to be feasible and is currently more widely used in some laboratories where clinical isolates or strains have been studied (Ko et al., 2019; Tafess et al., 2020). In this multicenter study, we evaluated the effectiveness of NanoTNGS in identifying *Mycobacterium tuberculosis* and the ability to determine resistance against drugs such as isoniazid, EMB, streptomycin, levofloxacin, and moxifloxacin directly using clinical specimens. Additionally, we compared the drug-resistance detection performance of rifampin between NanoTNGS and Xpert MTB/RIF.

The majority of the mutations associated with RIF resistance in the *rpoB* gene are located in an 81 bp region known as the RRDR, which encompasses codons 426–452 in *Mycobacterium tuberculosis* or 507–533 in *Escherichia coli* (Zaw et al., 2018; Hameed et al., 2022). Among those loci, codons 531, 526, and 516 exhibited important biological significance. In our study, the most common mutations detected in the *rpoB* gene were S531L (84 cases). Non-RRDR mutations in the *rpoB* gene have also been identified as additional molecular markers for predicting the fitness of clinical RIF-resistant *M. tuberculosis* strains (Ma et al., 2021). NanoTNGS successfully identified non-RRDR mutations in the *rpoB* gene, including F424V, D435G, H445D, S450W, S450L, E460G, I480V, and F505L (consensus numbering scheme of DNA-dependent RNA polymerase from *Escherichia coli*). Those mutations out of the RRDR region were associated with resistance to RIF in 11 samples confirmed by pDST. The controversial *rpo*B L511P mutation has been found in RIF-resistant and susceptible isolates (Jamieson et al., 2014; Ocheretina et al., 2015; Lin et al., 2021). The conflicting DST results for L511P depended on the culture method (MGIT 960 or agar plating) and sample types (Van Deun et al., 2009, 2013). In addition, double mutations were associated with phenotypic resistance to RIF (Ocheretina et al., 2015). In the present study, multiple mutations (P424V and L511P, G507fs and L511P, L511P and D516G, and L511P, D516G, and D516Y) in the *rpoB* gene were found in four phenotypically RIF-resistant samples, while no multiple mutations of *rpoB* were identified in phenotypically RIF-sensitive samples (Supplementary Table 5).

Mutations in the *katG* gene (*n* = 104) and *inhA* gene (*n* = 21) were found to be associated with the resistance phenotype to isoniazid (INH). Consistent with previous reports (Palomino and Martin, 2014; Ko et al., 2019), *katG* S315T and promoter region *inhA* c-15 t mutations were frequently observed in the INH-resistant gene. In our study, the most common INH-resistance mutation site was *katG* S315T (80.77%, 84/104), followed by *inhA* c-15 t (70.0%, 15/20) and *inhA* t-8c (30.0%, 6/21). A well-known mutation biomarker for high-level INH resistance is the *katG* gene mutation at codon 315. However, the interpretation of INH resistance-associated gene mutations should be more finely differentiated, as the c-15 t mutation in the *inhA* promoter confers not only low-level INH resistance but also high-level INH resistance (Rivière et al., 2020). It is crucial to confirm the mutation gene locus associated with drug resistance in those clinical settings. The assessment of *Mycobacterium tuberculosis* drug resistance by NanoTNGS may improve our understanding of the genotype–phenotype relationships to better guide clinical practice.

Regarding EMB resistance, codon 306 mutations in *embB* were found to mediate a large number of resistances (Telenti et al., 1997). Our study showed similar results that 46 out of 65 resistant samples showed variants in codon 306 (M306V, M306I, and M306L). It is worth noting that mutations in the *embB* gene (M306V and M306I) greatly reduced the specificity of EMB-resistance prediction due to overlapping MIC distributions with critical concentrations (Finci et al., 2022). Phenotypic DST for pyrazinamide was not routinely performed in clinical laboratories, partially due to the fact that the BACTEC MGIT 960 liquid culture assay is expensive, and pyrazinamide is active only in an acidic medium, in which some *Mycobacterium tuberculosis* isolates will fail to grow (Zhang and Mitchison, 2003; Maningi et al., 2015; Sodja et al., 2022). Moreover, previous research indicated that the *pncA* mutation could cause pyrazinamide resistance in *Mycobacterium tuberculosis* (Scorpio and Zhang, 1996). The mutation of *pncA* associated with pyrazinamide resistance showed high diversity, with a dispersed distribution in the codon. These mutations were not clustered (Tan et al., 2014) as *rpoB* genes associated with RIF resistance. The correlation between NanoTNGS and phenotypic susceptibility tests for pyrazinamide resistance detection should be studied in the future studies.

This study had several limitations. First, not all samples had pDST results for all drugs. However, pDST is not routinely conducted on newly diagnosed TB patients (Jouet et al., 2021). Moreover, we could not determine the association between mutations and resistance levels because minimum inhibitory concentration (MIC) testing was not performed. MIC resulting from the mutation will be detected in future studies, allowing for individualized drug dosing based on nanopore sequencing results.

## 5 Conclusion

To conclude, this study showed that NanoTNGS assays can be used to reliably detect *M. tuberculosis* drug resistance through targeted sequencing based on the Nanopore GridION platform. This rapid molecular diagnostic approach has potential applications in clinical practice and can facilitate the use of targeted sequencing as a cost-effective and faster diagnostic tool for managing TB. This epoch will greatly complement traditional methods, such as laboratory culture-based methods.

## Data availability statement

The raw sequence reads presented in this study can be found in online repositories. The names of the repository/repositories and accession number(s) can be found below: https://www.ncbi.nlm.nih.gov/, PRJNA1073707. Further inquiries can be directed to the corresponding author.

## Ethics statement

The studies involving humans were approved by the Medical Ethics Committee of Longtan Hospital of Guangxi Zhuang Autonomous Region. The studies were conducted in accordance with the local legislation and institutional requirements. Written informed consent for participation in this study was provided by the participants’ legal guardians/next of kin.

## Author contributions

AL: Conceptualization, Funding acquisition, Project administration, Writing – review & editing. SL: Conceptualization, Formal analysis, Writing – original draft. KL: Data curation, Investigation, Methodology, Writing – original draft. QZ: Data curation, Investigation, Methodology, Writing – original draft. JW: Data curation, Investigation, Methodology, Writing – original draft. JL: Data curation, Investigation, Methodology, Writing – original draft. CL: Data curation, Investigation, Methodology, Writing – original draft. XH: Data curation, Investigation, Methodology, Writing – original draft. CG: Data curation, Investigation, Methodology, Visualization, Writing – original draft. QS: Data curation, Investigation, Methodology, Visualization, Writing – original draft. HG: Conceptualization, Formal analysis, Writing – review & editing.
