# Supplementary material for: Rapid detection of multidrug resistance in tuberculosis using nanopore-based targeted next-generation sequencing: a multicenter, double-blind study
Source: Front Microbiol. 2024 Mar 1;15:1349715. doi: 10.3389/fmicb.2024.1349715 (PMC10940340; doi:10.3389/fmicb.2024.1349715)
Supplement: Supplementary file 1 [file Data_Sheet_1.zip › Supplementary material.docx]

Supplementary Material

**Rapid detection of multidrug resistance in tuberculosis using nanopore-based targeted next-generation sequencing: a multicentre, double-blind study**


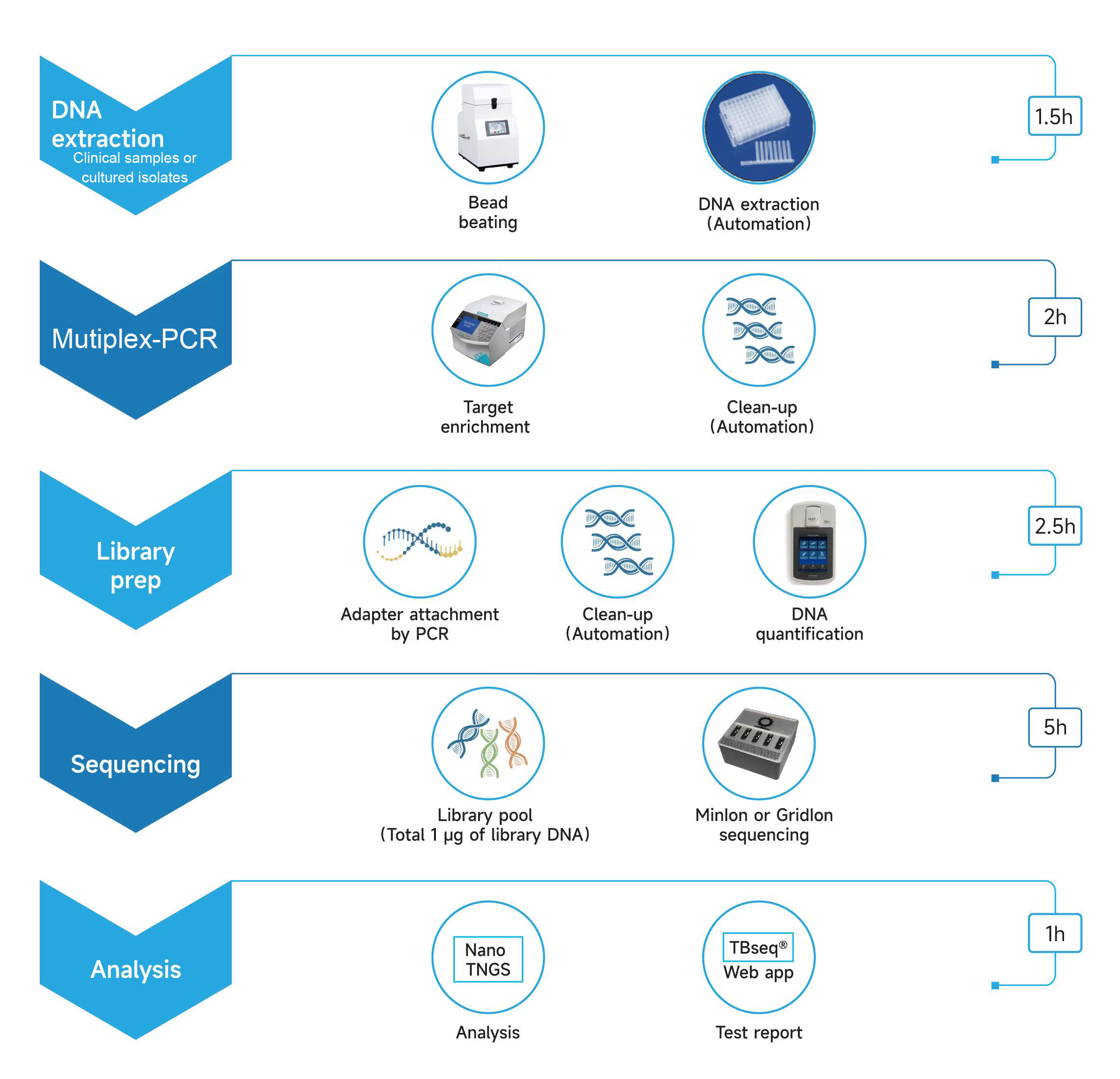


**Supplementary Figure 1.** Nanopore-based targeted next generation sequencing workflow. Estimated times provided for 32 tests sequenced on the Gridlon platform.


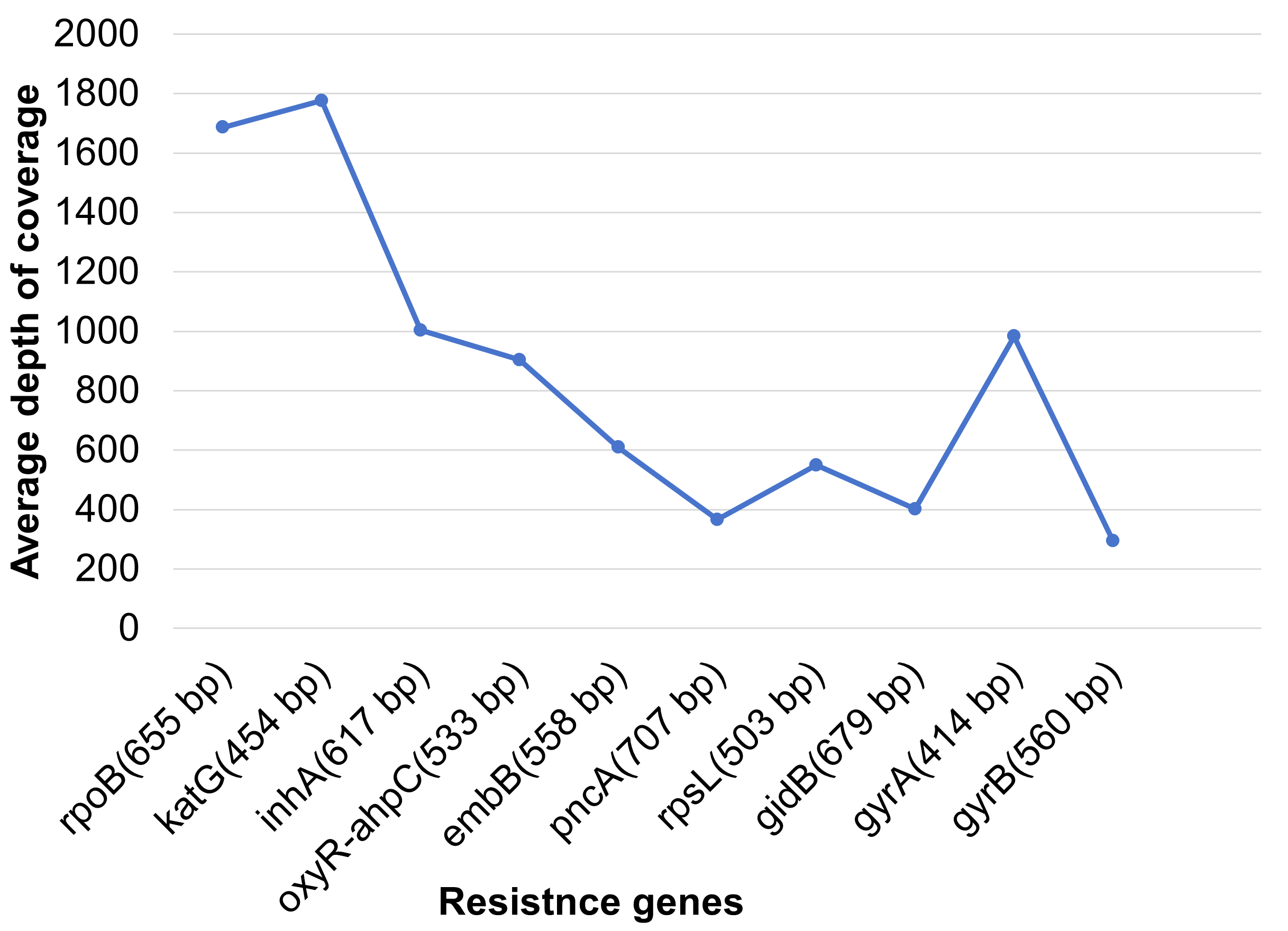


**Supplementary Figure 2.** The Average coverage depth of drug-resistant genes detected by NanoTNGS.


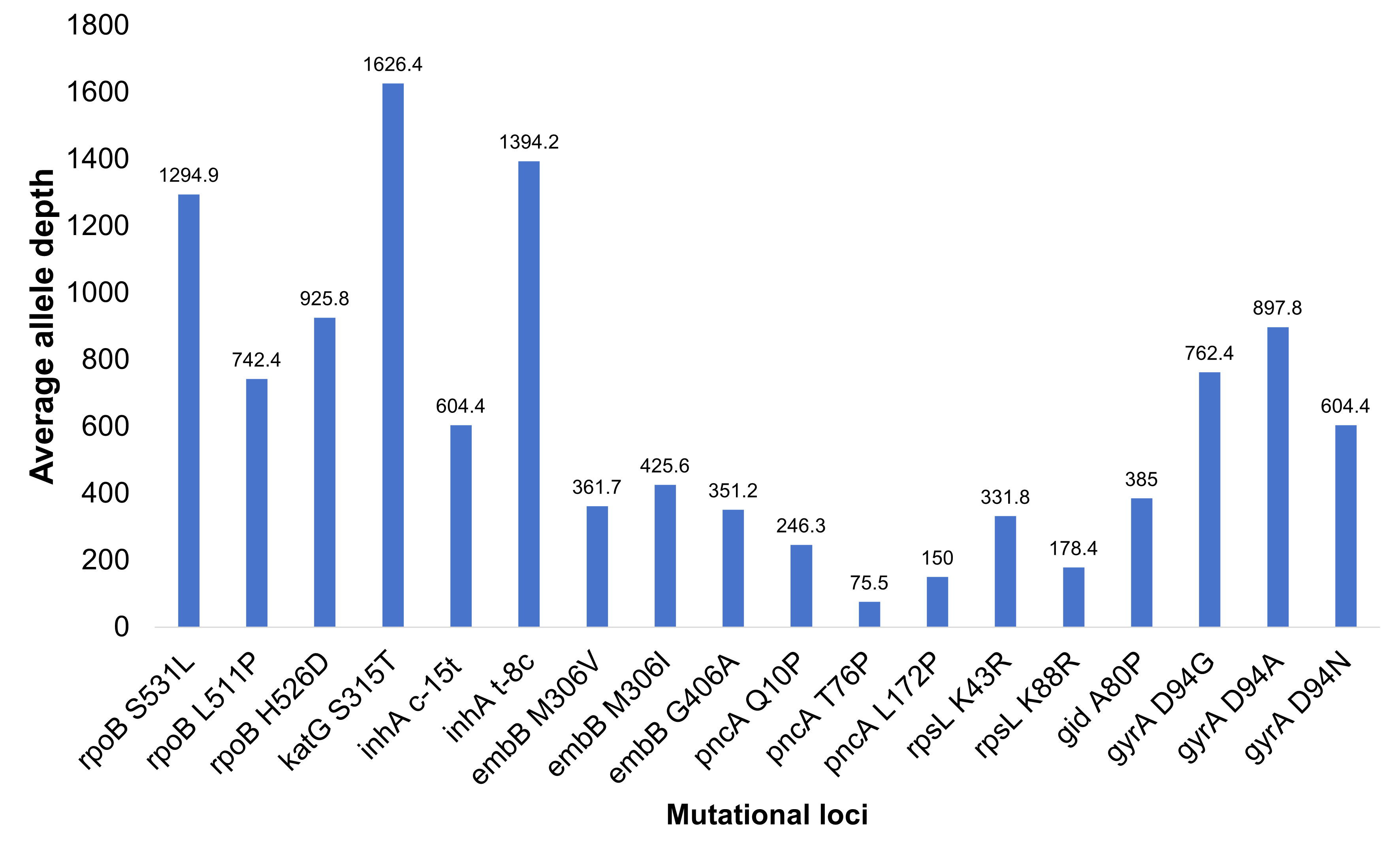


**Supplementary Figure 3.** The average allele depth of top 3 variants in detected resistance genes via NanoTNGS.

**Supplementary Table 1.** *Mycobacterium tuberculosis complex* gene regions targeted by nanopore-based targeted next generation sequencing.

| **Gene region** | **Drugs** | **Gene region** | **Drugs** |
| --- | --- | --- | --- |
| *rpoB* | Rifampicin | *eis, rrs* | Kanamycin |
| *ahpC, katG, inhA* | Isoniazid | *tlyA*, rrs* | Capreomycin |
| *pncA** | Pyrazinamide | *folC, thyA** | Para-aminosalicylic acid |
| *embB, embA* | Ethambutol | *ethA*, ahpC, inhA* | Ethionamide/protionamide |
| *rrs, rpsL*, gibB** | Streptomycin | *rv0678*, atpE** | Bedaquiline, clofazimine |
| *gyrA, gyrB* | Fluoroquinolones | *rplC** | Linezolid |
| *rrs* | Amikacin | *alr** | Cycloserine |

* full length genes.

**Supplementary Table 2.** Coverage depth of resistance genes detected by NanoTNGS.

**Supplementary Table 3.** The depth, allele depth and allele frequency of top 3 variants in detected resistance genes via NanoTNGS.

**Supplementary Table 4.** Bedaquiline, amikacin, kanamycin, and capreomycin resistance samples identified by NanoTNGS.

| **No.** | **Sample** | **Genotypic Resistant (NanoTNGS)** | **Gene** | **Nucleotide change** | **Amino acid change** | **Mutation rate (%)** | **pDST** |
| --- | --- | --- | --- | --- | --- | --- | --- |
| 1 | BALF | FQs  H  H  R  E  S  Bdq | *gyrA*  *katG*  *inhA*  *rpoB*  *embB*  *rpsL*  *Rv0678* | 281A>G  944G>C  -15C>T  1592C>T  916A>G  128A>G  281G>A | D94G  S315T  -  S531L  M306V  K43R  R94Q | 100  100  99.57  100  100  98.51  25 | RHES Ofx resistance |
| 2 | Sputum | S  Am, Km, Cm  FQs  R  H  E  R | *rrs*  *rrs*  *gyrA*  *rpoB*  *inhA*  *embB*  *rpoB* | 514A>C  1401A>G  271T>C  1379A>G  -15C>T  1217G>C  1576C>T | -  -  S91P  E460G  -  G406A  H526Y | 100  99.86  99.83  99.81  99.76  98.1  96.26 | RHES Ofx Km PAS resistance |
| 3 | BALF | E  H  R  FQs  Am, Km, Cm | *embB*  *inhA*  *rpoB*  *gyrA*  *rrs* | 918G>A  -15C>T  1577A>G  281A>G  1401A>G | M306I  -  H526R  D94G  - | 93.07  99.45  99.8  99.88  99.89 | RHE Ofx resistance |
| 4 | Sputum | H  R  S  Am, Km, Cm  Z  H | *katG*  *rpoB*  *rpsL*  *rrs*  *pncA*  *inhA* | 944G>C  1592C>T  128A>G  1401A>G  20T>G  -8T>C | S315T  S531L  K43R  -  V7G  - | 100  100  100  100  99.17  99.09 | RHS Am resistance |
| 5 | Sputum | Am, Km, Cm  H  H  Z  R | *rrs*  *katG*  *katG*  *pncA*  *rpoB* | 1401A>G  1147T>C  836G>A  83C>A  1576C>A | -  S383P  G279D  A28D  H526N | 97.67  97.83  99.21  99.39  99.66 | H Am Mfx Km Cfz resistance |
| 6 | Sputum | S  R  Am, Km, Cm  H  Z | *rpsL*  *rpoB*  *rrs*  *katG*  *pncA* | 128A>G  1577A>G  1401A>G  944G>C  515T>C | K43R  H526R  -  S315T  L172P | 98.18  99.04  99.59  100  100 | RHS Km resistance |

Abbreviation: BALF, bronchoalveolar lavage fluid. pDST, phenotypic drug susceptibility testing. R, rifampicin. H, isoniazid. Z, pyrazinamide. E, ethambutol. S, streptomycin. Mfx, moxifloxacin. Ofx, ofloxacin. Am, amikacin. Km, kanamycin. Cm, capreomycin. FQs, fluoroquinolones. Bdq, bedaquiline.

**Supplementary Table 5.** Samples carrying the mutations L511P in the *rpoB* gene.

| **No.** | **Sample** | **Gene** | **Nucleotide change** | **Amino acid change** | **Xpert MTB/RIF** | **pDST** |
| --- | --- | --- | --- | --- | --- | --- |
| 1 | BALF | *rpoB*  *ahpC* | 1532T>C  -52C>T | L511P  - | R resistance | H resistance |
| 2 | Sputum | *rpoB*  *rpoB*  *embB* | 1270T>G  1532T>C  1217G>C | F424V  L511P  G406A | R resistance | RH resistance |
| 3 | BALF | *rpoB*  *katG*  *gyrA* | 1532T>C  943A>G  271T>C | L511P  S315G  S91P | R resistance | ND |
| 4 | BALF | *rpoB* | 1532T>C | L511P | R resistance | ND |
| 5 | Sputum | *embB*  *rpoB*  *rpoB*  *katG* | 1216G>T  1532T>C  1578C>G  944G>C | G406C  L511P  H526N  S315T | R resistance | ND |
| 6 | Sputum | *embB*  *rpsL*  *katG*  *rpoB*  *rpoB* | 916A>G  128A>G  944G>C  1520delG  1532T>C | M306V  K43R  S315T  G507fs  L511P | R resistance | RHS resistance |
| 7 | BALF | *katG*  *rpoB*  *katG* | 945C>A  1532T>C  943A>G | S315R  L511P  S315G | R resistance | Sensitive |
| 8 | BALF | *ahpC*  *rpoB* | -72C>T  1532T>C | -  L511P | R resistance | H resistance |
| 9 | Sputum | *rpoB* | 1532T>C | L511P | R resistance | Sensitive |
| 10 | Sputum | *rpoB* | 1532T>C | L511P | R resistance | R resistance |
| 11 | BALF | *rpoB* | 1532T>C | L511P | R resistance | R resistance |
| 12 | Sputum | *rpsL*  *rpoB*  *rpoB*  *katG*  *embB*  *gyrA* | 263A>G  1532T>C  1547A>G  944G>C  1217G>C  281A>C | K88R  L511P  D516G  S315T  G406A  D94A | R resistance | RHES Ofx resistance |
| 13 | BALF | *katG*  *rpoB* | 944G>C  1532T>C | S315T  L511P | ND | H resistance |
| 14 | BALF | *rpoB* | 1532T>C | L511P | ND | S resistance |
| 15 | Sputum | *gyrA*  *rpoB*  *rpoB*  *gyrA*  *rpoB* | 269C>T  1546G>T  1547A>G  281A>G  1532T>C | A90V  D516Y  D516G  D94G  L511P | ND | R resistance |
| 16 | BALF | *katG*  *rpoB*  *embB* | 944G>C  1532T>C  916A>G | S315T  L511P  M306V | R resistance | HE resistance |

Abbreviation: BALF, bronchoalveolar lavage fluid. pDST, phenotypic drug susceptibility testing. R, rifampicin. H, isoniazid. E, ethambutol. S, streptomycin.. Ofx, ofloxacin.
